# Gold Nanomaterial Uptake from Soil Is Not Increased by Arbuscular Mycorrhizal Colonization of *Solanum Lycopersicum* (Tomato)

**DOI:** 10.3390/nano6040068

**Published:** 2016-04-13

**Authors:** Jonathan D. Judy, Jason K. Kirby, Mike J. McLaughlin, Timothy Cavagnaro, Paul M. Bertsch

**Affiliations:** 1 Commonwealth Science and Industry Research Organization (CSIRO) Land and Water, Waite Road, PMB 2, Urrbrae 5064, South Australia, Australia; jason.kirby@csiro.au (J.K.K.); mike.mclaughlin@csiro.au (M.J.M.); 2 School of Agriculture, Food and Wine, University of Adelaide, Waite Campus, PMB 1, Glen Osmond 5064, South Australia, Australia; timothy.cavagnaro@adelaide.edu.au; 3 Center for the Environmental Implications for Nanotechnology, Duke University, Durham, NC 27708, USA; paul.bertsch@csiro.au; 4 Commonwealth Science and Industry Research Organization (CSIRO), Land and Water, Ecosciences Precinct, 41 Boggo Road, Dutton Park 4102, Queensland, Australia

**Keywords:** nanotoxicology, nanotechnology, ecotoxicology, risk assessment

## Abstract

Bioaccumulation of engineered nanomaterials (ENMs) by plants has been demonstrated in numerous studies over the past 5–10 years. However, the overwhelming majority of these studies were conducted using hydroponic systems and the degree to which the addition of the biological and chemical components present in the soil might fundamentally alter the potential of plant bioaccumulation of ENMs is unclear. Here, we used two genotypes of *Solanum lycopersicum* (tomato), reduced mycorrhizal colonization (*rmc*), a mutant which does not allow arbuscular mycorrhizal fungi (AMF) colonization, and its progenitor, 76R, to examine how colonization by AMF alters trends of gold ENM bioaccumulation from a natural soil. Gold was taken up and bioaccumulated by plants of both genotypes. Gold concentrations were significantly higher in the *rmc* treatment although this was likely attributable to the large differences in biomass between the 76R and *rmc* plants. Regardless, there was little evidence that AMF played a significant role in trafficking Au ENMs into the plants. Furthermore, despite very low NH_4_NO_3_ extractable Au concentrations, Au accumulated at the root-soil interface. Although this observation would seem to suggest that ENMs may have potential to influence this particularly biologically active and important soil compartment, we observed no evidence of this here, as the 76R plants developed a robust AMF symbiosis despite accumulation of Au ENMs at the rhizoplane.

## 1. Introduction

Over the past decade, many studies have reported plant uptake and translocation of engineered nanomaterials (ENMs), largely from hydroponic and culture media [1,2,3]. Although studies investigating ENM uptake from soil have become more common [4,5,6,7,8,9], many knowledge gaps remain regarding ENM bioavailability and toxicity in soil. A natural soil environment will have many important chemical and biological components that a hydroponic exposure setting does not possess and the possibility exists that the addition of these components may fundamentally alter phytotoxicity and/or plant bioavailability.

Plants form beneficial symbioses in soil with microorganisms including arbuscular mycorrhizal fungi (AMF). AMF spores produce hyphae which penetrate plant roots and serve as a conduit for the symbiotic exchange of inorganic ions and carbohydrates. However, the initiation of this relationship alters physiological barriers to ENM uptake, as the penetration of the root by AMF hyphae forms a site through which ENMs may possibly enter the root, as well as potentially acting as a pathway through which the AMF may traffic ENMs from the soil into the plant [10].

Recent studies have begun to examine how AMF might influence uptake of ENMs and how ENMs might impact plant-mycorrhizal relationships [6,11,12,13]. Watts-Williams *et al.* reported that nanoparticulate ZnO had no negative effect on AMF colonization of tomato in a 4:1 sand/soil mixture and that mycorrhizal plants took up the same amount of Zn as did non-mycorrhizal plants [11]. Judy *et al.* examined the effects of Ag ENMs, Ag_2_S ENMs, and Ag^+^ on AMF colonization of tomato in a biosolids-amended sandy loam [6]. At 100 mg·kg^−1^ of each of these treatments, AMF colonization was almost completely inhibited by the Ag^+^ and Ag ENM treatments, whereas the Ag_2_S ENM treatment, which is more analogous to the Ag ENM species that would be discharged into the environment [14], had no significant effect. Shoot Ag concentrations were lower in the Ag_2_S treatment than the Ag ENM treatments, suggesting that the increased AMF colonization of the Ag_2_S treated plants did not result in greater bioaccumulation of Ag.

However, examining how AMF might affect ENM uptake was not among the objectives of these studies, and the data collected that might address this question are limited. As a result, the degree to which AMF may alter and potentially increase plant uptake of ENMs in a natural soil is not clear. Considering 80% of vascular plants form symbioses with AMF, this is important question that needs to be addressed [10]. Therefore, in this study, we use the *Solanum lycopersicum* (tomato) genotypes 76R (which allows AMF infection) and *rmc* (which does not allow AMF infection) to examine uptake of gold (Au) ENMs from soil and to investigate the degree to which infection by AMF affects the translocation of ENMs from the soil into plant roots. Citrate-stabilized gold ENMs are used here as a model ENM species with which to probe bioaccumulation and translocation without the challenges associated with chemical transformations.

## 2. Results

**Figure 1:**
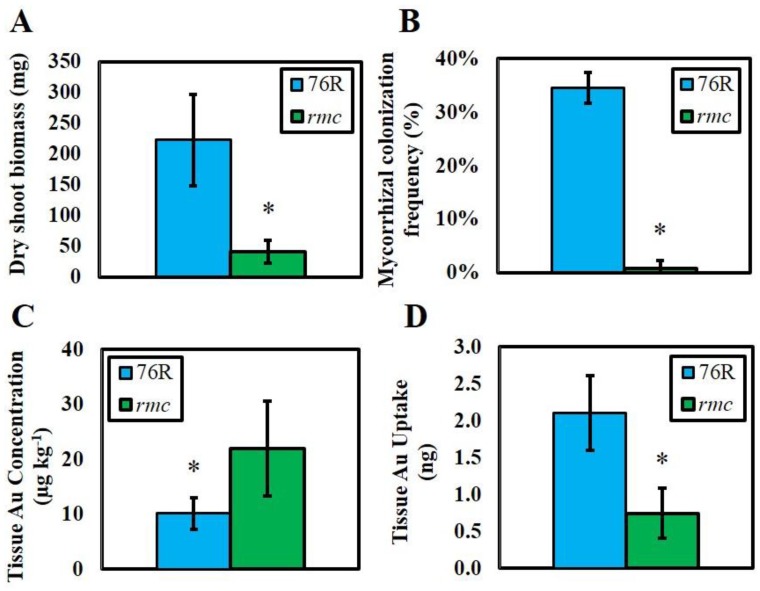
Dry shoot biomass (**A**); mycorrhizal colonization frequency (**B**); shoot Au concentrations (**C**); and shoot Au uptake (**D**) measured in 76R and *rmc* tomato plants. Error bars represent standard deviation. The * indicates a significant difference at α ≤ 0.01 as determined by a either a 2-sided *T*-test (concentration and uptake) or a Mann-Whitney *U*-test (biomass and colonization).

Dry shoot biomass produced by *rmc* plants was significantly lower (*p* ≤ 0.05) than that produced by the 76R plants (Figure 1A). Analysis of concentrations and uptake of Au into shoot tissue indicated that both the 76R and *rmc* plants accumulated Au despite virtually zero AMF colonization of the roots in the *rmc* plants (Figure 1B). Plants from the 76R treatment accumulated a significantly larger mass of Au (*p* < 0.01) whereas the *rmc* plants contained a significantly higher concentration of Au (*p* < 0.01). Shoot tissue concentrations ranged from 6–14 µg Au·kg^−1^ for the 76R treatment and 11–30 µg Au·kg^−1^ for the *rmc* plants, whereas tissue uptake ranged from 1.3–2.6 ng for the 76R plants and 0.6–1.1 ng for the *rmc* treatment (Figure 1C, D).

The concentration of NH_4_NO_3_ extractable Au in soil was 87.5 ± 21.1 µg Au·kg^−1^ (*n* = 3), less than 0.5% of the added 25 mg Au·kg^−1^ soil Au ENM concentration. Measurement of the concentration of dissolved Au in ENM background, as determined by high-speed centrifugation, estimated that dissolved Au was 0.008% ± 0.0004% of the total Au present in the ENM treatments. These measurements indicate that the majority of the extractable Au measured via this procedure was, at least initially, in the form of ENMs and not a product of ENM dissolution.

**Figure 2:**
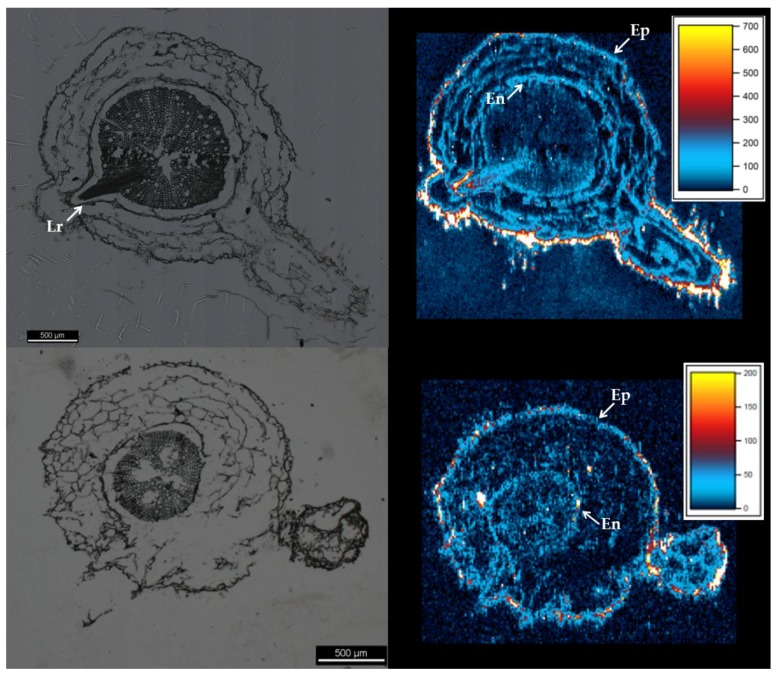
Micrographs (**left**) and laser ablation inductively-coupled plasma mass spectrometry (LA-ICP-MS) maps (**right**) of root cross-sections collected from root samples from (**top**) 76R and (**bottom**) *rmc* tomato plants. Color bars inset in LA-ICP-MS maps show relationship between counts per second (CPS) and color for each map. Ep = epidermis. En = endodermis. Lr = lateral root.

Laser ablation inductively-coupled plasma mass spectrometry (LA-ICP-MS) heat maps from root cross-sections indicate that Au accumulated at the root epidermis. However, hot spots were detected within the root cross sections, indicating that a small amount of ENMs were transferred into the interior of the root, a finding consistent with the low but detectable Au measured in the shoots. The majority of ENMs that penetrated the root epidermis concentrated at the root endodermis within cross-sections from both the 76R and *rmc* plants, although hotspots did occur at other locations within the root cortex. The cross-sections analysed also included incipient lateral roots, which Au appeared to accumulate around even before the lateral root had penetrated the root exterior (Figure 2). It was not possible to discern whether Au ENMs were present in the hyphae of AMF in the roots of 76R plants.

## 3. Discussion

Tissue analysis and LA-ICP-MS maps of roots were consistent in showing that a small amount of Au was taken up and translocated to the shoots by the plants of both genotypes. However, even though uptake is detected and reported here, the amounts of Au bioaccumulated were extremely small, suggesting that the bioavailability of the Au ENMs tested here was low. What uptake pathway or mechanism was responsible for the bioaccumulated ENMs observed here is unclear. The localization of Au at the epidermis of roots from both genotypes indicates that uptake was impeded. However, the detection of Au with the root cortex, at the root endodermis, and within the shoots reveals that these barriers were not able to completely prohibit uptake. Research reporting plant uptake of ENMs has suggested apoplastic uptake, endocytotic uptake, and uptake via wounding as possible pathways of ENM uptake, among others [15,16,17]. Reports have begun to accumulate suggesting that apoplastic transport may be the preferential pathway of ENMs uptake by plants [16,18]. The Au evident within the LA-ICP-MS maps presented here seems to be largely associated with cell walls, which although consistent with apoplastic transport, may also be an artifact of sample preparation, *i.e.*, that the ENMs within the plant root cells relocated to cell walls during sample fixation. The LA-ICP-MS imaging here also suggests that incipient root development zones may present less robust barriers to uptake, and may possibly account for some component of measured bioaccumulation.

Although measurements of shoot concentrations and uptake of Au revealed significant differences between cultivars, this trend was likely driven largely by the difference in dry shoot biomass (Figure 1A). Considering this strong influence, there is little evidence within our data for major changes in the bioavailability of Au ENMs in response to AMF colonization. The lack of fundamental differences in Au bioaccumulation between the two tomato genotypes suggests that AMF colonization of plant roots either had no or not enough impact to affect the affect the association of soil-delivered ENMs with plants. This finding differs from a report by Whiteside *et al*. (2009) which reported AMF uptake and transfer of quantum dots (QDs) in mycorrhizal roots of *Poa annua* (annual bluegrass) [13]. In this earlier study, plants were exposed to QDs in a 1:1 sand: vermiculite mixture. QDs coated with an organic nitrogen (N) coating were taken up whereas bare QDs were not, which the authors’ speculated was the result of active uptake processes initiated by the AMF in order to obtain organic N. The possibility exists that the surface charge or the nutritive composition of the coating molecule may have similarly affected the results in the current study, and a systematic investigation regarding how surface chemistry affects ENM uptake by AMF colonized plants may be warranted. Similarly, higher resolution studies using methods such as transmission electron microscopy (TEM) that could potentially examine ENMs within AMF hyphae would also be useful.

## 4. Materials and Methods

### 4.1. Nanomaterials

**Figure 3:**
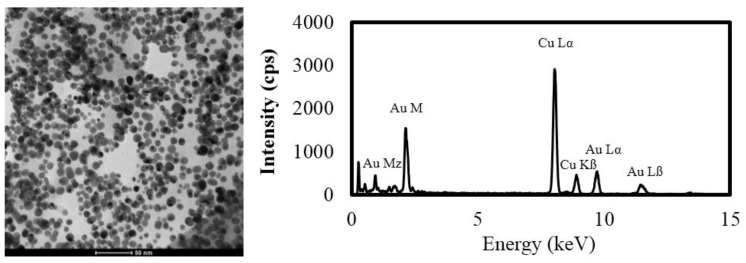
TEM (transmission electron microscopy) micrograph and energy-dispersive X-ray spectroscopy (EDS) spectrum characterizing gold engineered nanomaterials (ENMs). Copper detected is result of the use of Cu TEM grids. cps: counts per second.

As previously mentioned, Au ENMs were used as a model ENM species as it is resistant to chemical transformation and therefore useful as a probe from uptake and translocation. Gold ENMs were synthesized via citrate-stabilized aqueous reduction [19]. Two liters of 1 mM HAuCl_3_·3H_2_O was heated to 100 °C and 160 mL of 38.8 mM Na_3_-citrate was added. The suspension was boiled for 15 min and then allowed to cool at room temperature. Resulting ENMs were characterized using TEM/energy-dispersive X-ray spectroscopy (EDS), dynamic light scattering (DLS), and phase analysis light scattering (PALS; Figure 3 and Table 1). DLS and PALS analyses were performed using a Nano-ZS Zetasizer (Malvern Instruments Ltd, Worcestershire, UK) and electrophoretic mobilities were converted to zeta potentials using the Hückel approximation. TEM analysis was conducted on ENMs mounted on 200 mesh copper TEM grids with a Tecnai G2 Spirit (FEI, Hillsboro, Oregon, MA, USA). Particle size was determined from measurements >100 particles from at least 3 TEM micrographs using ImageJ software (National Institutes of Health, Bethesda, MD, USA; Table 1). Background dissolved Au was estimated by centrifuging samples of Au ENMs at 18,407× *g* for 6 h (designed to sediment Au particles <3 nm, minimum size observed in TEM micrographs) and subsequently acidifying samples of the supernatant to 2% hydrochloric acid for later analysis using an Agilent 7700x inductively coupled plasma mass spectrometer (ICP-MS; Santa Clara, CA, USA) [1].

### 4.2. Plant Exposure

Seeds from the 76R and *rmc* genotypes, isolated and described in Barker *et al.* (1998), were pre-germinated on moistened paper towels in the dark for one week prior to planting [20]. The day before planting, Au ENMs suspended in deionized water (DI) were added to Black Point sandy loam at a rate of 25 mg·kg^−1^ and the soil wetted to 60% water holding capacity (WHC), mixed thoroughly, and allowed to equilibrate overnight. Black Point soil is an alkaline (pH 8.3, as determined in a 1:5 soil/water mix) sandy loam with cation exchange capacity of 20.5 cmol·kg^−1^ and 1.4% total carbon. The morning after ENM addition, one seed was planted into each 1 L pot containing 1 kg of soil. There were 5–6 pots per treatment. Plants were watered with DI three times per week. Starting 24 days after planting, equal volumes of 10% Hoagland’s solution were added to each pot weekly, with additions becoming biweekly after 38 days [21]. Plants were grown using a 12 h day (23 °C) and 12 h night (18 °C) light cycle at 65% relative humidity for 8 weeks.

### 4.3. Au Extractions from Soil

Samples of the media were collected at the start of the experiment, oven-dried at 60 °C and shaken in 25 mL 1 M NH_4_NO_3_ for 2 h [22]. Tubes were centrifuged at 2778× *g* for 20 min, after which the supernatant was collected and filtered through a 0.45 µm filter. Extracts were then digested in 7.5 mL concentrated HCl and 2.5 mL HNO_3_ by microwave assisted digestion using United States Environmental Protection Agency Method 3051A [23]. Extracts were subsequently analysed by inductively coupled plasma mass spectrometry (ICP-MS).

### 4.4. Quantification of Mycorrhizal Colonization

Roots were collected, cleared of cellular contents using 10% KOH, and stained using the ink and vinegar method [24]. Mycorrhizal colonization frequency was quantified using the gridline intersection method [25].

### 4.5. Shoot Au Analysis

Shoot tissues were weighed directly into glass tubes, after which samples were digested at 115 °C for 4 h in 2 mL of concentrated HNO_3_ and allowed to cool. The following morning, 6 mL of concentrated HCl was added and samples were heated to 60 °C and allowed to cool. As there is no widely available plant tissue reference material containing Au, two laboratory fortified matrix (LFM) samples were created by spiking oven-dried tobacco tissue with two separate certified Au solutions (High-Purity Standards, Charleston, SC, USA), oven drying the slurries, and grinding resulting pellets with a mortar and pestle. Recoveries using these LFMs were 91.0 ± 2.3% (*n* = 3) and 94.2 ± 0.9% (*n* = 3). Root tissues were allocated to the quantification of mycorrhizal colonization and LA-ICP-MS and due to this, as well as the difficulty distinguishing surface adsorbed ENMs from bioaccumulated ENMs, roots were not analysed for bulk Au content.

As concentrations of Au in plant tissues were expected to be very low, ICP-MS analysis was calibrated across a very low analytical range (0.001–2 µg·L^−1^). Furthermore, a rigorously derived method detection limit (MDL) was calculated based upon the analysis of ten 0.05 µg·L^−1^ LFM solution samples as recommended in US Department of the Interior *Open File Report (OFR) 99-193*, resulting in 99% confidence that detected Au concentrations were greater than zero [26]. The result of these measures was an 8.6 ng·L^−1^ analytical MDL and a tissue uptake detection limit of 0.57 ng·plant^−1^. For statistical analyses, ½ MDL was substituted for one value that remained below the detection limit (BDL) for the *rmc* treatment.

### 4.6. Root Sectioning and Analysis by LA-ICP-MS

Roots from selected plants were collected and stored in 50% ethanol prior to cryosectioning at The University of South Australia. Cryosections were collected from the tap root just below the shoot and were 12 µm thick. Sections were mapped using laser ablation inductively coupled mass spectrometry (LA-ICP-MS) using a Resonetics M-50-LR 193-nm Excimer laser microprobe coupled to an Agilent 7700cx quadrupole ICP-MS housed at Adelaide Microscopy (Adelaide, South Australia, Australia). The M-50 utilizes a two-volume small volume ablation cell designed by Laurin Technic Pty (Narrabundah, Australian Capital Territory, Australia) [27]. Ablation was performed in an atmosphere of UHP He (0.7 L·min^−1^). Upon exiting the cell the aerosol is mixed with Ar (0.83 L·min^−1^) immediately after the ablation cell, after which the mix is passed through a pulse-homogenizing device prior to introduction into the ICP-MS.

The laser beam had a spot size of 15 μm and was rastered across the samples in a grid with a scan speed of 30 μm·s^−1^. The spacing between lines was maintained at 15 μm to match the size of the laser beam. The beam had a laser repetition of 10 Hz and an energy output of 80 mJ which resulted in an energy density of ~4 J·cm^−2^ at the target. A set of 7 elements were analyzed with the dwell time for all masses set to 0.03 s except for C12 which used a dwell time of 0.01 s. A 30 s background acquisition was acquired at the start of every raster. Additionally, a delay of 15 s was used after each line to allow for signal wash-out, gas stabilization and data processing. Rasters were also collected on United States Geological Survey (USGS) reference glasses National Institute of Standards and Technology (NIST) standard reference material (SRM) 610 at the start and end of each map.

Data were processed using the free-ware software program Iolite [28]. To correct for drift during mapping runs, standards were analyzed immediately before and after the run to assess drift and if present, data were corrected by applying a linear fit between the two standard analyses. The average background signal was subtracted from the corresponding raster and all rasters were compiled into a two dimensional image.

### 4.7. Statistics

Statistical analyses were performed using SAS 9.4. Data were tested for normality and homoscedasticity using Shapiro-Wilk’s and Bartlett’s tests. Normal data and non-normal data that could be log transformed to provide normality were analyzed via a two-sided 2-sample *T*-test. Data that could not be normalized were analyzed via a Mann-Whitney *U*-test.

## 5. Conclusions

The LA-ICP-MS data reported here demonstrate that ENMs added to a natural soil have the potential to migrate to and/or concentrate at the rhizoplane, despite seemingly low bioavailability in soil (as determined by chemical extraction). This finding suggests that the Au ENMs used here may have been stable to some degree in soil pore water in the soil used here, consistent with other recent work [29]. However, it is also possible that the ENMs observed accumulated on the exterior of the roots were those that the plant root encountered in the soil immediately surrounding the roots. Regardless, this accumulation has implications for the toxicity as well as potential agronomic use of other ENMs that release free ions (e.g., Ag and ZnO ENMs). As many critical biochemical processes and plant-microorganism relationships are initiated at the rhizoplane, the accumulation of ENMs in this zone suggests there may be more potential for plants to be affected by soil accumulation of ENMs than traditional methods of estimating metal bioavailability, such as the tissue analysis and metal extractions conducted here, might indicate. However, the data collected here demonstrate robust AMF colonization of the 76R plants compared to typical values in unexposed plants [6,11], despite rhizoplane accumulation. This finding, which is similar to other recent work examining the effects of Au ENMs on rhizosphere biota [30], suggests that AMF colonization may be relatively resistant to potential effects induced by at least Au ENM accumulation at the rhizoplane over the time periods tested here.

## Figures and Tables

**Table 1:** Nanomaterial characterization data (mean ± standard deviation, unless otherwise noted). Hydrodynamic diameter (Z-average diameter), measured by dynamic light scattering, based on intensity weighted size distribution measurements. TEM: transmission electron microscopy.

Treatment	Z-Average Diameter (nm)	Polydispersivity Index	TEM Diameter (nm)	TEM Range (nm)	Zeta Potential (mv ± zeta deviation)
Au ENMs	26.5 ± 4.9	0.29 ± 0.1	9.9 ± 2.7	3.5–17.8	−58.0 ± 5.9

## Abbreviations

The following abbreviations are used in this manuscript:

CSIRO	Commonwealth Scientific and Industrial Research Organization
ENM	Engineered nanomaterial
AMF	Arbuscular mycorrhizal fungi
LFM	Laboratory fortified matrix
*rmc*	Reduced mycorrhizal colonization (tomato genotype)
LA-ICP-MS	Laser ablation inductively-coupled plasma mass spectrometry
